# Improved prediction of critical residues for protein function based on network and phylogenetic analyses

**DOI:** 10.1186/1471-2105-6-213

**Published:** 2005-08-26

**Authors:** Boris Thibert, Dale E Bredesen, Gabriel del Rio

**Affiliations:** 1 Buck Institute For Age Research, 8001 Redwood Blvd, Novato, CA 94945, USA; 2 University of California, San Francisco, San Francisco, CA 94143, USA; 3 Instituto de Fisiología Celular, UNAM, Circuito Exterior, Ciudad Universitaria, 04510, México, D.F

## Abstract

**Background:**

Phylogenetic approaches are commonly used to predict which amino acid residues are critical to the function of a given protein. However, such approaches display inherent limitations, such as the requirement for identification of multiple homologues of the protein under consideration. Therefore, complementary or alternative approaches for the prediction of critical residues would be desirable. Network analyses have been used in the modelling of many complex biological systems, but only very recently have they been used to predict critical residues from a protein's three-dimensional structure. Here we compare a couple of phylogenetic approaches to several different network-based methods for the prediction of critical residues, and show that a combination of one phylogenetic method and one network-based method is superior to other methods previously employed.

**Results:**

We associate a network with each member of a set of proteins for which the three-dimensional structure is known and the critical residues have been previously determined experimentally. We show that several network-based centrality measurements (*connectivity*, *2-connectivity*, *closeness centrality*, *betweenness and cluster coefficient*) accurately detect residues critical for the protein's function. Phylogenetic approaches render predictions as reliable as the network-based measurements, although, interestingly, the two general approaches tend to predict different sets of critical residues. Hence we propose a hybrid method that is composed of one network-based calculation – the *closeness centrality *– and one phylogenetic approach – the *Conseq *server. This hybrid approach predicts critical residues more accurately than the other methods tested here.

**Conclusion:**

We show that network analysis can be used to improve the prediction of amino acids critical for protein function, when utilized in combination with phylogenetic approaches. It is proposed that such improvement is due to the complementary nature of these approaches: network-based methods tend to predict as critical those residues that are highly connected and internal (i.e., non-surface), although some surface residues are indeed identified as critical by network analyses; whereas residues chosen by phylogenetic approaches display a lower overall probability of being surface inaccessible.

## Background

This article deals with the problem of predicting critical amino acid residues for a given protein's function, i.e., those residues that, if mutated, result in a loss of protein function by the lack of proper folding and/or the inability to perform a biochemical function (hereafter referred to simply as *critical residues*). A common approach to this problem consists of aligning multiple orthologous protein sequences, and predicting as critical residues the most highly conserved ones. This approach is known as the phylogenetic approach, and requires that a significant number of distinct protein sequences be aligned. This approach also assumes that the aligned sequences from different species serve the same function in the different species. However, for an increasing number of proteins studied by the Structural Genomic Consortium, a different trend is observed: for a given protein there may be a lack of a significant number of homologous sequences, whereas the three-dimensional structure (3D structure) of the protein of interest may already have been described. For this set of proteins, having an accurate way to identify critical residues in the absence of known orthologues – i.e., simply from the protein's three-dimensional structure – may be valuable. Identifying critical residues from protein structures can also be extended to those proteins for which there are a significant number of homologous sequences [1]. Furthermore, the prediction of critical residues from protein structures has been shown to be useful in validating protein structure predictions [2,3].

Over the past few decades, network analyses have been used to model diverse systems such as the World Wide Web, social systems, and biological systems (e.g., protein-protein interactions or the cellular metabolic network) [4]. Only very recently has such an approach been used to predict critical amino acids from protein structures [[5,6] and [7]].

By definition, a network is composed of two sets: a set of vertices and a set of edges (an edge being a pair of connected vertices). A protein can be modelled in a network as follows: each vertex of the network represents an amino acid residue, and each edge represents a chemical interaction between any two amino acid residues. In the particular case in which the 3D structure of the protein is known, it can be assumed, for the sake of the model, that there is a chemical interaction between two amino acids if they are sufficiently close to one another (less than 5 Angstroms apart in our case; see Methods). This gives a way of building a network of amino acid interactions [[5,6] and [7]], with the obvious caveat that the accuracy of such a model will be affected by the accuracy of the assumption of chemical interactions between different residues.

Previous studies have related specific centrality measurements of these amino acid interaction networks to the tolerance for replacement (i.e., critical nature) of the various amino acids within the network. In particular, the *most traversed *vertices (also referred as vertices with the greatest *betweenness*) have been shown to be important for folding [5] and for the functions of the modelled proteins [7]. These vertices, when detected from several structures for a given protein, relate more accurately with critical residues than the most conserved residues detected from some phylogenetic approaches [7]. Hence, *betweenness*, a network centrality measurement, is associated with the capacity of any given amino acid residue to play a critical role in a protein's function. These results indicate that critical residues are central to the interactions among the residues in a protein structure. However, whether or not the experimentally determined critical residues are best predicted by measures of *betweenness*, as opposed to any other centrality measurement, has not been tested. Evaluation of other centrality measurements may also help to understand the significance of the observed relationship.

In the current work we compare five different network centrality measurements, applying them to the study of the structure/function relationship of proteins. Previous studies on the structure/function relationship of proteins have shown that critical residues tend to display specific geometrical characteristics, such as distorted dihedral angles or central location, buried within the proteins' cores ([8] and references therein). However, whether these geometrical properties are related to network centrality (*e.g., betweenness*) has not been studied. In this work, we analyze some of these relationships, as well.

It is possible to define several centrality measurements at a vertex *p *of a network. Some of these centrality measurements have already been used for modelling different systems. One of the most direct and widely used centrality measurements is the *connectivity c*_*p *_([9], for example), which is the number of neighbours of *p *(*i.e*., the number of vertices that share an edge with *p*). Another, the *clustering coefficient *[9], defined by 2*n*_*p*_/*c*_*p*_(*c*_*p *_- 1), where *n*_*p *_is the number of connections between the neighbours of *p*, estimates the degree of interconnectivity among the neighbours of *p*. These two measurements are local, in the sense that their values only depend on the vertex *p *and on its neighbours.

There are also global centrality measurements assignable to every vertex in a network (*i.e*., whose values depend on the structure of the whole network). Many of these are based on the notion of shortest path. We say that a path connects two vertices *p *and *q *if there exists a sequence of edges that connect *p *and *q*. The length of a path is its number of edges. The distance *l*(*p*, *q*) between *p *and *q *is the length of the shortest path connecting *p *and *q*. Hence, one of these global centrality measurements is the *closeness centrality – *also referred as *chemical distance *[[10,11] and [6]] – and is defined as the inverse of the average distance value between *p *and all the other vertices of the network. Another, the *eccentricity *[12], is the inverse of the maximum distance from *p *to any other vertex. We can also define the *betweenness *at a vertex *p *[5,7] as the number of all shortest paths connecting two vertices of the network that pass through *p *normalized by the total number of paired vertices.

Here, we show that *closeness centrality *correlates more accurately with critical residues than *betweenness *or any other centrality measurement tested. Also, we show that the sets of critical residues predicted by any of the centrality measurements (e.g., *betweenness*, *closeness centrality*) do not completely overlap with those based on phylogeny. Hence, we present evidence that combined predictions based on *closeness centrality *and phylogeny improve the predictions achieved by any of these approaches alone. Finally, we observe that the critical residues detected by centrality measurements correlate with low-accessibility solvent residues, although these are not exclusively in the protein's core. These results are used to suggest an explanation for the improvement achieved by the combined approach to detect critical residues.

## Results and discussion

### Five methods based on network analyses

We propose and compare five different centrality measurements to predict critical amino acids from a protein structure: *connectivity*, *cluster coefficient*, *closeness centrality*, *betweenness *and *2-connectivity*. The *2-connectivity *at a vertex *p *is the number of vertices connected to *p *with fewer than 2 edges. This new measurement is local and can be seen as a generalization of the *connectivity*.

Let us describe now how we proceed to determine the critical residues from one of these centrality measurements, for example the *closeness centrality *(the method described for *closeness centrality *can be extended to the other four measurements). We assume that the amino acids that have the *largest closeness centrality *are critical for the function of the protein. Since the set of predicted critical residues depends on the cut-off value used to define the "*largest closeness centrality*", we evaluated all possible cut-off values.

Hence, we define five methods to predict critical residues, each one based on a different centrality measurement. For each, the set of critical amino acids is defined to be composed of the vertices:

• Method 1: with the largest *closeness centrality*,

• Method 2: with the largest *betweenness*,

• Method 3: with the largest *2-connectivity*,

• Method 4: with the largest *connectivity*,

• Method 5: with the smallest *cluster coefficient*.

We compared these methods on a set of five well-characterized proteins in terms of their 3D structures and biochemical identification of critical residues. These include TEM1 beta-lactamase, T4 lysozyme and HIV-1 protease [[13,14] and [15]]. Exhaustive mutagenesis and analysis has been utilized to identify every residue critical for the functioning of these three proteins, and therefore they serve as excellent test proteins for the computational methods described here. We also include Barnase [16] and the bacteriophage f1 gene V protein [17]. Two differences are relevant for these two proteins with respect to the previous set of three proteins mentioned before: the number of homologous sequences available is reduced and the sensitivity of the biological assay is augmented. For the beta-lactamase and the protease of the HIV-1, more than 40 unique homologous sequences were found by the *Conseq *server, while for the bacteriophage f1 gene V protein only 6 homologous sequences were found on the *Hssp *database. For Barnase, fewer than 5 homologous sequences were found by the *Conseq *server and 20 homologous sequences were found within the *Hssp *database (see Methods). Although our choice of these proteins was not simply because of the relative lack of homologous sequences, this limitation of the current experimental data serves to illustrate the point that a method that does not require a large number of homologous sequences is desirable as a complement to phylogenetic analyses. Finally, the sensitivity of the biological assay employed to identify critical residues in Barnase (inactive mutants were defined as those having < 1% of wild-type activity) was much higher than the one employed for the other four proteins, thus reducing the total number of critical residues detected experimentally.

In order to evaluate the quality of our predictions, we use the sensitivity and the specificity statistical measurements. However, one of these two criteria alone is not sufficient to compare the different methods (see Methods). Therefore, in order to obtain a single value to evaluate the predictions of our methods that includes both sensitivity and specificity, we propose three different strategies:

1. For a given centrality measurement and cut-off value, we calculate the error *dist*(*centr. meas., cut-off*) (see Methods). This error considers simultaneously the specificity and the sensitivity. It is small if both the sensitivity and the specificity are good. This error allows us to sort all the predictions obtained by any centrality measurement/cut-off value used (this is used in Table 1 and 4).

**Table 1:** Sorting of the methods according to the sensitivity and specificity criteria (5 proteins set)

**Method**	**Proportion of predicted residues**	**Error**	**Sen.**	**Spe.**
Union set (10)	38%	**0,41**	71%	73%
2-connectivity (3)	42%	**0.43**	74%	67%
Conseq	42%	**0,44**	76%	67%
Closeness (1)	38%	**0.44**	68%	71%
Intersection set (9)	38%	**0,44**	68%	71%
Connectivity (4)	38%	**0,45**	67%	71%
1/cluster (5)	38%	**0.48**	63%	70%
Betweenness (2)	38%	**0.50**	62%	69%
1/eccentricity	45%	**0.53**	68%	60%
Hssp	38%	**0.54**	62%	66

For every method, results are obtained in average over five 3D structures: 1HIV, 2LZM, 1BTL, 1A2P and 1GVP. Numbers are obtained the following way: For a given *cut-off *value (between 0% and 100% of residues that are predicted), we calculate the average (over the five proteins) of the *dist*(*method*, *cut-off*) error, the sensitivity and the specificity. This is done for several cut-off values. Then we choose the cut-off for which the average of the *dist*(*method*, *cut-off*) error is minimal. Note that in most of the cases, the error is the smallest if we predict around 40% of the amino acids to be essential. Furthermore the smallest error for *1/eccentricity *is worse than the smallest errors for the five other centrality measurements (1 to 5).

**Table 4:** Sorting of different methods according to the sensitivity and specificity criteria (128 proteins set)

**Method**	**Proportion of amino acids predicted**	**Error**	**Sen.**	**Spe.**
Hssp	24%	**0,41**	72%	77%
Conseq	27%	**0,44**	71%	74%
Union set (10)	25%	**0,50**	61%	76%
Closeness	36%	**0,56**	64%	64%
Intersection set (9)	32%	**0,58**	60%	68%
Betweenness (2)	33%	**0,58**	58%	67%
2-connectivity (3)	37%	**0,60**	60%	64%
1/cluster (5)	49%	**0,63**	68%	51%
1/eccentricity	24%	**0,64**	44%	77%
Connectivity (4)	49%	**0,65**	65%	51%

Results are obtained in average other the set of 128 proteins. Numbers are obtained by calculating the average as in Table 1.

**Figure 1:**
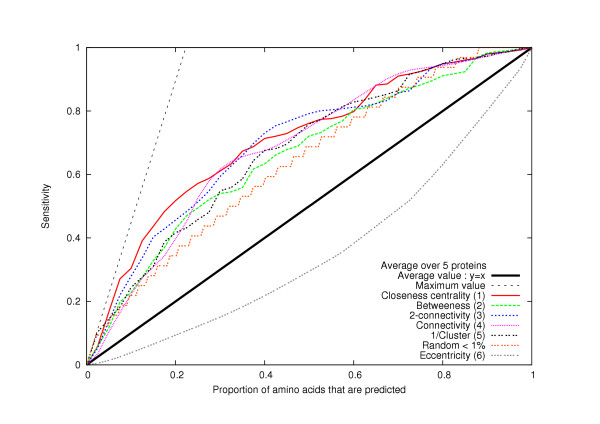
**Comparison of 6 different network-based methods for essential amino acids prediction**. For each method (Methods 1–6 and "Random <1%") the abscissa x is the proportion of amino acids predicted (by the appropriate centrality measurement) and y is the proportion of essential amino acids predicted (*i.e*. sensitivity). For example, x = 0.2 means that we select 20% of the amino acids. If we consider the *closeness centrality*, it means that we select 20% of the amino acids that have the largest *closeness centrality*. We can notice in this case that we select around 38% of the essential amino acids. The slide of the "Maximum value" curve is the proportion of amino acids that are essential. All the curves have to be under this curve. The better a method is, the closer to the "Maximum value" its associated curve is. A method is better than random if its curve is over the "Average value: y = x" curve. A method is worse than random if its curve is under the "Average value: y = x" curve. The calculations (of the curves 1–6) are made as an average over the five networks representing HIV-1 protease, TEM1 beta-lactamase, T4 lysozyme, Barnase and bacteriophage f1 gene V protein. The curve "Random <1%" only depends on the average number of amino acids of the proteins. There is a probability less than 1% that a random selection of the amino acids will produce a curve over the "Random <1%" curve.

**Figure 2:**
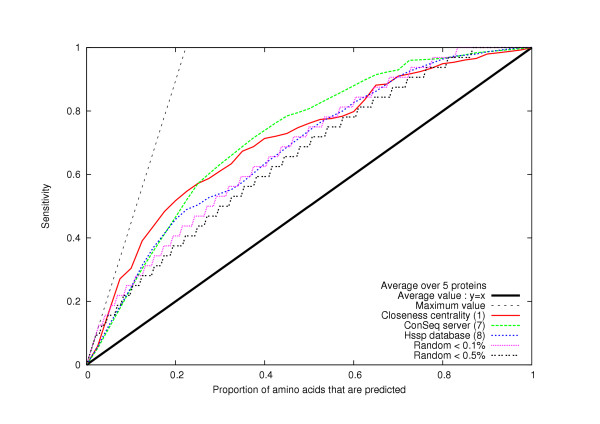
**Comparison between phylogenetic, random and network methods**. We compare method 1 (based on *closeness centrality*) with two phylogenetic methods (method 7 -*Hssp *database- and method 8 -*Conseg *server-) and with a random selection of the amino acids. There is a probability less than 1% (*resp*. 0.5%) that a random selection of the amino acids will produce a curve over the "Random <1%" curve (*resp*. "Random <0.5%" curve). We notice that if we predict between 10% and 50% of the amino acids, there is a probability less than 0.5% that a random selection of the amino acids will give better results than methods 1 and 7. The calculations are made as an average over the five networks representing HIV-1 protease, TEM1 beta-lactamase, T4 lysozyme, Barnase and the bacteriophage f1 gene V protein.

**Figure 3:**
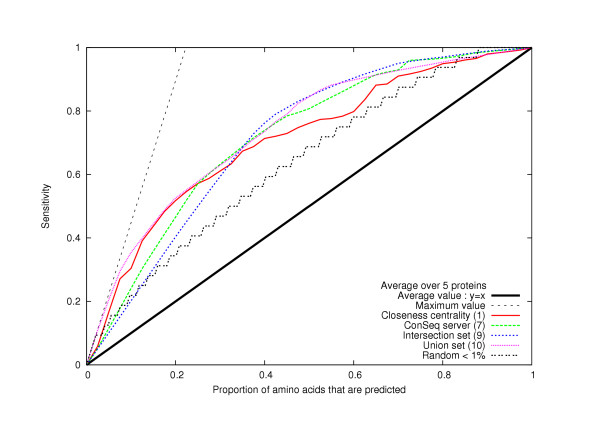
**A new method for essential amino acid prediction**. We compare the new method (based on the two sets of amino acids *Union *and *Intersection*) with method 1 (based on *closeness centrality*) and the *Conseq *server. This method is better than the others: indeed, if we predict fewer than 35% of the amino acids, then the curve (9) is, over most of that range, the highest one; if we predict more than 60% of the amino acids, the curve (10) is, over most of that range, the highest. The calculations are made as an average over the five networks representing HIV-1 protease, TEM1 beta-lactamase, T4 lysozyme, Barnase and the bacteriophage f1 gene V protein.

2. For a given centrality measurement and cut-off value, we also compare the sensitivity value to the proportion of predicted amino acids (number of predicted residues/total number of residues in a protein). This is relevant, because the sensitivity is greater than the proportion of predicted amino acids if and only if the method is better than random (see Methods, Figs. 1, 2 and 3). Furthermore, by plotting the sensitivity versus the proportion of predicted amino acids (see Figs 1, 2 and 3), we can compare all the methods with all of their different cut-off values.

3. We also compare the specificity values of the different methods when the sensitivity is equal to 50% (see Tables 5 and 6).

**Table 5:** Specificity value for each method at the sensitivity = 50% level (5 proteins set)

**Method**	**Specificity**	**Proportion of predicted residues**
Union set (10)	89%	18%
Conseq	85%	22%
Intersection set (9)	85%	23%
Closeness	85%	22%
Connectivity (4)	85%	22%
2-connectivity (3)	83%	28%
Betweenness	80%	27%
1/cluster (5)	80%	27%
Hssp	80%	24%
1/eccentricity	76%	30%

For every method, results are obtained in average over the set of five proteins. Numbers are obtained by calculating the average as in Table 1. For each method, we indicate the value of the specificity and the proportion of residues that are predicted when the sensitivity is equal to 50%.

**Table 6:** Specificity value for each method at the sensitivity = 50% level (128 proteins set)

**Method**	**Specificity**	**Proportion of predicted residues**
Union set (10)	87%	15%
Conseq	87%	14%
Intersection set (9)	77%	24%
Closeness	77%	23%
Connectivity (4)	65%	35%
2-connectivity (3)	73%	28%
Betweenness	74%	26%
1/cluster (5)	68%	33%
Hssp	87%	14%
1/eccentricity	68%	32%

Results are obtained in average over the set of 128 proteins. Numbers are obtained by calculating the average as in Table 1. For each method, we indicate the value of the specificity and the proportion of residues that are predicted when the sensitivity is equal to 50%.

We show that the five methods give better results than a random prediction of critical residues, at any given cut-off value selected (in Fig. 1, the curves of Methods 1 to 5 represent the different methods and are always more accurate than the "random selection" that is represented by the line "y = x"). That is, if we randomly select between 10% and 75% of the amino acids in a given protein, there is a probability of less than 1% to have better results than with Methods 1–4 (indeed, the curves of Methods 1 to 4 are above the "Random < 1%" curve for 0,1 < x < 0,75).

Furthermore, if we predict as critical residues fewer than 30% of the protein's amino acids, the best method is based on the global centrality measurement *closeness centrality *(Method 1). Alternatively, if we predict as critical residues 30% to 55% of the protein's amino acids, the method based on the local centrality measurement *2-connectivity *(Method 3) gives the best results (See Fig. 1 and Table 1). Method 1 (based on *closeness centrality*) always gives better results than Method 2 (based on *betweennes *(see Fig. 1).

We also tried other network centrality measurements (*e.g., eccentricity and 1/eccenticity*), However, the results obtained indicated much less accuracy than with these five methods: the method using the *eccentricity *measurement is worse than random (see Fig. 1) and the method using *1/eccentricity *measurement is worse than Methods 1–5 (see Table 1).

Our results improve the reliability of predictions based on the network centrality measurement *betweenness *described in [7], in which one structure was used to identify critical residues. This is probably due to the fact that we considered several cut-off values. For example, if we want to maximize both the specificity and the sensitivity for the predictions based on *betweenness*, we need to predict around 40% of a protein's amino acids (Table 1), while in [7] less than 20% of a protein's amino acids were predicted as critical.

### Different centrality measurements do not recognize the same set of critical amino acids

The comparison of the level of accuracy in predicting critical residues of the five network centrality-based methods and two phylogenetic approaches reveals that these phylogenetic methods (see Methods) are as reliable as the best network centrality-based methods (Fig. 2). We analyzed whether the sets of critical residues identified by any of these approaches display overlap. If there is little or no overlap, then combining the predictions from these methods may improve the reliability of their individual predictions.

**Figure 4:**
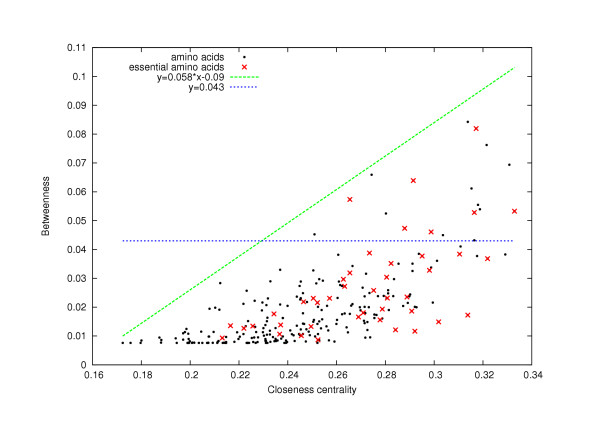
**Relationship between the *betweenness *and the *closeness centrality *for TEM1 beta-lactamase**. For each amino acid of TEM1 beta-lactamase, we plot the *betweenness *versus the *closeness centrality*. We notice that the points are not approximately disposed along a "natural curve", which suggests that there is no natural "functional relationship" between these two centrality measurements. Therefore, we cannot deduce one centrality measurement from the other one. However, we notice that the cloud of points is located under the curve "y = 0.058x-0.09". This implies that the set of amino acids with the highest *betwenness *is included in a set of amino acids with highest *closeness centrality *(for a given cut-off).

There is no strong "functional relationship" between any two different network-based centrality measurements. In other words, we cannot express precisely one of these properties as a mathematical function of any other properties. For example, plotting the *betweenness *versus *closeness centrality *(Fig. 4) shows a cloud of points that are not disposed on a curve. More generally, we do not observe any strong "functional relationship" between any two of these network-based centrality measurements: the *connectivity*, the *cluster coefficient*, the *eccentricity*, the *closeness centrality*, the *betweenness *and the *2-connectivity*. Hence, the centrality-based methods (Methods 1–5) may not predict the same set of critical amino acids.

Alternatively, looking at a less rigorous relationship among centrality measurements, we did observe some overlap. As shown in Fig. 4, the critical amino acids detected by two network centrality-based measurements present some overlap: if the *betweenness *of an amino acid is high, then its *closeness centrality *is typically high, as well. Hence, the set of vertices with high *betweenness *is completely included in a set of vertices with high *closeness centrality*. A similar trend is also observed for other centrality measurements, as expected from the fact that these all share some common factors (*e.g*., shortest path, see Background section).

**Figure 5:**
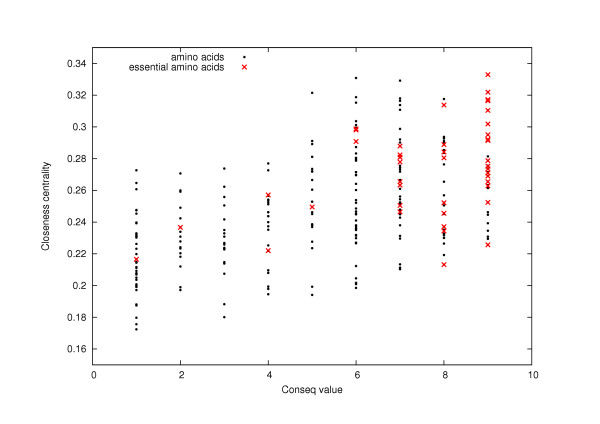
**Relationship between the *closeness centrality *and the *Conseq *value for TEM1 beta-lactamase**. For each amino acid of TEM1 beta-lactamase, we plot the *closeness centrality *versus the *Conseq *value. We notice that the points are not approximately disposed along a "natural curve", which suggests that there is no strong natural "functional relationship" between these two centrality measurements and that we cannot deduce precisely one centrality measurement from the other one.

There is also no strong "functional relationship" between the set of amino acids predicted with any of the five network methods and the set of amino acids predicted with phylogenetic approaches. This also implies that a network method and a phylogenetic method do not predict the same set of residues, although they both present similar reliability in their abilities to predict critical residues. For example, Fig. 5 shows that the score from one of the phylogenetic approaches used (*Conseq *server, [18]) is not related to the *closeness centrality *score (data shown only for 1BTL). Therefore, since these two methods predict with the best confidence critical residues and do not predict the same set of amino acids, a combination of the two methods is expected to improve these results (at least to improve sensitivity; whether the overall accuracy will be improved depends on the effect on specificity as well as sensitivity).

### An improved method using the *Conseq server *and the *closeness centrality*

We propose a new method that improves the accuracy of the predictions achieved by the phylogenetic approaches tested here (see Methods) or the five methods based on network analyses. This method is based on combining critical residues predicted by *closeness centrality *and by one phylogenetic approach (*ConSeq *server, [18]). The method is now briefly described.

For a given number *k*, the set *Union *is obtained by combining both the *k *amino acids that have the largest *Conseq *value and the *k *amino acids that have the largest *closeness centrality *value. The set *Intersection *is obtained by taking the common amino acids between the *k *amino acids that have the largest *Conseq *value with the *k *amino acids that have the largest *closeness centrality *value.

If we predict less than 35% of a protein's amino acids to be critical for protein function, then the set *Intersection *gives better results than both phylogenetic approaches and centrality-based methods; if we predict more than 60% of the amino acid residues, the *Union *set gives the best results (Fig. 3). Furthermore, the sensitivity and the specificity considered simultaneously are better for the *Union *set than for the other methods (Table 1). If we compare the different methods for a sensitivity value of 50%, we notice that the *Union *set is also giving the best results (Table 5). Therefore, we have a method that improves both the phylogenetic methods and the centrality-based methods.

### Testing the method in a different set of proteins

In order to evaluate our combined method in a different set of proteins, we used a set of 128 proteins (see Methods). These proteins all have their three-dimensional structures solved, and information is available about some but not all of their critical residues (information available from the SITE annotation in the PDB files). This set was used to evaluate the reliability of the predictions of the *closeness centrality *(see Table 2). We observed as with the previous set of proteins, that for a sensitivity value of 50%, the *Union *set predicts more true critical residues than phylogenetic approaches (i.e., presents a higher specificity value) (see Table 6).

**Table 2:** Estimated Sensitivity and Specificity for *closeness centrality*

**Protein(s)**	**Sensitivity**	**Specificity**
1BTL	67.4%	72.7%
1HIV	58.7%	83.0%
2LZM	68.3%	78.8%
1A2P	75.0%	89.4%
1GVP	87.5%	64.5%
Average(lBTL, lHIV, 2LZM, lA2P, lGVP)	71.4%	77.7%
Average(128 proteins set)	63.9%	64.1%

The sensitivity and specificity values are reported for each individual protein (1BTL, 1HIV, 2LZM, 1A2P and 1GVP), and the average values obtained for these proteins and the set of 128 proteins. These sensitivity and specificity values correspond to the best cut-off value of the error (i.e. smallest *dist*(*centr. meas., cut-off*)) estimated for the *closeness centrality*. Results in average are obtained by taking the best cut-off value for every protein (and not by taking a fixed cut-off as in Table 1).

To compare closeness centrality against phylogenetic approaches, we noticed that the SITE annotation in this 128 proteins, mostly included residues annotated to be involved in catalysis (218 residues), ligand binding (156 residues) and/or metal-binding (273 residues) sites (see Table 3). Many of the metal binding sites were identified from crystallization conditions, as opposed to be truly involved in any biochemical function. For instance, 77 out of 273 residues involved in metal binding sites participated in catalysis. Our method identified with similar or better reliability than phylogenetic methods active site residues, ligand binding sites and metal binding sites involved in catalysis (see Table 3). Overall, phylogenetic methods predict with better reliability the set of annotated sites for these 128 proteins, mainly because these methods detected better metal binding sites than our method.

**Table 3:** Type of residues predicted by network and phylogenetic analyses

**SITE**	**Closeness Centrality**	**HSSP**	**ConSeq**
Metal binding	75	126	134
Metal binding in active site	52	24	38
Ligand binding	57	63	59
Active site	110	104	116

The number of predicted residues that were annotated as SITE in 128 selected proteins is reported for columns Closeness Centrality, HSSP and ConSeq. These last two columns correspond to the results obtained with phylogenetic methods (see Methods section). Four classes of SITE annotations were distinguished and included in column SITE.

### Relationship between central amino acids and protein surface-accessible area

**Figure 6:**
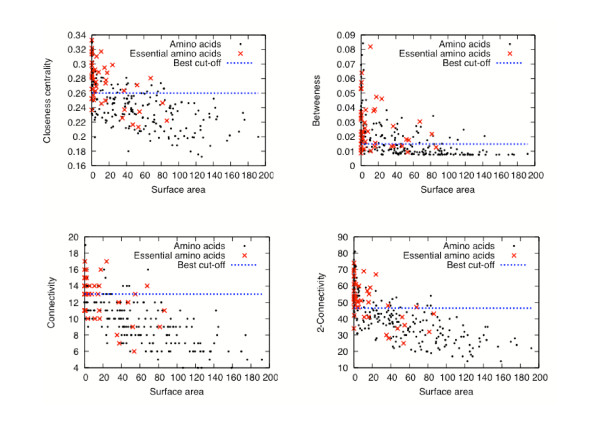
**Relationship between some network-based centrality measurements and the surface area for TEM1 beta-lactamase**. For each amino acid of TEM1 beta-lactamase (1BTL), we plot four network centrality measurements (*closeness centrality*, *betweenness*, *connectivity *and *2-connectivity*) versus the *surface area*. The "best cut-off curve corresponds to the cut-off that minimizes the *dist*(*centr. meas., cut-off*) error. In other words, for the four centrality measurements, the best result is obtained by predicting an amino acid to be critical if it is above this line (the corresponding set of predicted amino minimizes the error *dist*(*centr. meas.,cut-off*)).

Looking for an explanation for why the combination of phylogeny with network centrality renders improved results in the prediction of critical residues, we decided to analyze the nature of the residues detected by these two approaches. It has been previously shown that critical amino acids present special geometrical properties [8]. For instance, critical residues have been proposed to have a tendency to be in low surface-accessibility areas [19]. Since proteins are compact molecules and the methods described here aim to detect residues central to a protein's amino acid interactions, it is expected that central residues may be at the protein's core (*i.e*., present low-accessibility surfaces). Indeed, TEM1 beta-lactamase, HIV-1 protease, T4 lysozyme, Barnase and bacteriophage f1 gene V protein have their critical residues at a low-surface area (data are shown for TEM1 beta-lactamase in Fig. 6): if we assume that residues above 100 square Angstroms of surface accessibility are exposed, and buried otherwise, all critical residues for this protein are buried (low-accessibility area), as well as all central residues. Furthermore, phylogenetically highly conserved residues also present this trend: conserved residues have low surface accessibility.

**Figure 7:**
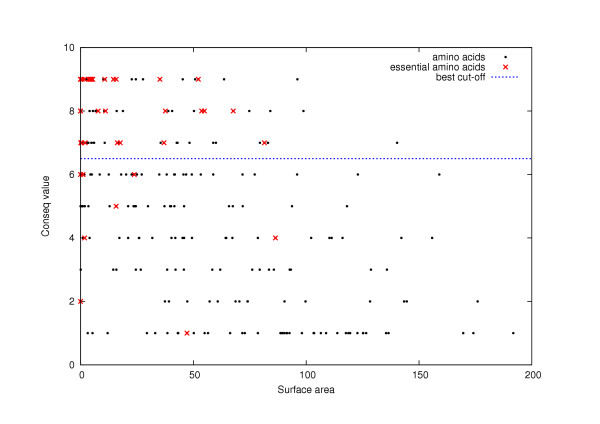
**Relationship between the *Conseq *value and the surface area for TEM1 beta-lactamase**. For each amino acid of TEM1 beta-lactamase (1BTL), we plot the *Conseq *value versus the *surface area*. The best cut-off for the *Conseq *value is given by the "best cut-off curve: the set of residues above this curve is the set that minimizes the error *dist*(*conseq value, cut-off*).

However, it is clear that the surface accessibility measurement alone is not a good predictor of critical residues (see Figs. 6 and 7). Also, it is important to note that the cut-off value chosen to define buried residues is an arbitrary one. In this sense, critical residues are not all "buried" inside a protein; some of them tend to be "exposed" (*e.g*., catalytic sites, cofactor binding sites). For instance, the critical residues detected in the five proteins by *closeness centrality *include active site residues that are not core residues (see Table 3). In any case, changing the criterion that defines core residues does not change the conclusions reached here: network centrality measurements are good predictors of the criticality of an amino acid in protein structure and function.

On the other hand, there is a relationship between the surface accessibility and the centrality measurement for a given residue (data are shown for 1BTL in Fig. 6): the most central residues tend to have a low-accessibility surface. This relationship is not observed for the conserved residues (data only shown for 1BTL in Fig. 7). Hence, in order to explain our results showing centrality measurements as good predictors of critical residues, we propose that these measurements represent more accurately the property that establishes a residue as one critical for protein function: critical residues are central to the residue-residue interactions and these tend to have low-accessibility surfaces.

While revising this manuscript, we became aware that Amitai and colleagues [20] reported that *closeness centrality *was effective in identifying critical residues from protein structures in a different set of proteins than ours. This may constitute an additional validation that centrality is a new feature of protein structures that is related to the function of residues in a protein. However, we cannot compare directly our results with theirs. The main difference is the procedure used to build the network of contacts in these two studies. Based on this difference, Amitai and colleagues reported a correlation between conserved residues and central ones, a feature we did not observe. Another difference is that the specificity of the method reported by Amitai and colleagues (<10%) is much lower than ours (>70%). Also, the sensitivity of both methods differs (Amitai and col. ~40%, ours >70%). However, Amitai and colleagues found that *closeness centrality *is related to low surface accessibility, just as we did.

## Conclusion

We compared several methods for prediction of critical residues for a given protein function. In the case in which the protein cannot be aligned with a significant number of homologues, we provide five network-based methods that require the proteins' 3D structures but do not require homologous proteins. Although these five methods do not predict the same sets of critical residues, they all give results much better than random.

In the case in which a protein with known 3D structure has enough protein sequence homologues, phylogenetic approaches are as reliable as the five network-based methods. More importantly, there is little overlap in the set of predicted critical residues by any compared method. Hence we propose a new method based on both a network centrality measurement (*e.g., closeness centrality*) and a phylogeny approach (*e.g., Conseq *server, [18]) to predict critical residues for protein function based on the 3D structure of proteins and multiple sequence alignments. This hybrid approach improves upon the results of any of the methods compared here.

We observed that there is a trend in plotting centrality measurements and surface accessibility: the most central residues are also those with least likelihood of surface exposure. Such a trend is not as striking for conservation scores and surface accessibility. Since critical residues tend to be more within or near the core (as noted above, central residues identify not only core residues but also active site residues), we propose that the improvement achieved by combining phylogeny with network centrality measurements is due to the complementary nature of these two approaches.

## Methods

### Set of proteins

We studied three proteins: TEM1 beta-lactamase, HIV-1 protease and T4 lysozyme. We chose this set of proteins because these have been systematically mutated and their 3D structures are available. The Protein Data Bank (PDB) files used for each of these three proteins are respectively 1BTL, 1HIV and 2LZM [[13,14] and [15]]. For this set of proteins, saturation single-point mutation experiments were performed, thus allowing for the identification of every single residue being important for each protein's function. In our study, a critical residue is defined as one important for the function of a protein as a whole (i.e., folding and biochemical function). For these three proteins, the biological assay identified critical residues as those mutants with less than 20% of activity with respect to the wild-type protein [[13,14] and [15]].

Two more proteins were analyzed in this study: Barnase [16] and the bacteriophage f1 gene V protein [17], for in these two cases the proteins have been systematically mutated and their 3D structures are available. The Protein Data Bank (PDB) files used for each of these three proteins are respectively 1A2P and 1GVP. In the case of the 1GVP protein, the biological assays evaluated the activity in both *E. coli *survival and the bacteriophage f1 propagation ability. So, two different sets of critical residues were identified for these two separate essays. Here we considered as critical residues those that were critical in both assays.

Finally, we used a set of 128 proteins from the PDB database that contained information about critical residues (*i.e*., SITE annotations on the PDB file) and were defined as representative folds in the FSSP database. The 128 PDB names used in this set are: 1a3c, 1a6q, 1a7j, 1aac, 1ac5, 1ah7, 1ak1, 1ako, 1amj, 1an8, 1apq, 1arv, 1atg, 1auz, 1ayl, 1ayx, 1az9, 1b64, 1bag, 1bdb, 1bea, 1bfd, 1bia, 1bif, 1bix, 1bk0, 1bli, 1bn5, 1bor, 1boy, 1bp1, 1bqk, 1brt, 1btl, 1c25, 1ca1, 1cby, 1cex, 1cfb, 1chc, 1chd, 1csh, 1ctn, 1ctt, 1cvl, 1dmr, 1drw, 1dxy, 1ecl, 1eh2, 1emn, 1esl, 1eut, 1far, 1fnc, 1gca, 1htn, 1hyt, 1iba, 1ido, 1iow, 1iyu, 1kcw, 1kpf, 1lam, 1lay, 1lbu, 1lgr, 1lml, 1lox, 1mfs, 1mla, 1mrp, 1mup, 1nif, 1opc, 1pda, 1pdc, 1pfo, 1phd, 1phm, 1pii, 1pkp, 1poa, 1poc, 1rfs, 1rie, 1rkd, 1rlw, 1skf, 1snc, 1sra, 1thx, 1uch, 1uox, 1ush, 1whi, 1wod, 1xbd, 1xpa, 1ytw, 2abk, 2adr, 2af8, 2cba, 2cmd, 2dkb, 2dri, 2fha, 2fua, 2liv, 2mcm, 2mnr, 2rn2, 2sas, 2vil, 3dfr, 3dni, 3ebx, 3gcb, 3pte, 3ssi, 3tgl, 4enl, 4icb, 4pah, 5eat and 7rsa.

### Network associated to a protein

We created one network per protein structure in the following way: we calculated the distance *d*(*a*_1_, *a*_2_) between two amino acids *a*_1 _and *a*_2 _by:



where *a*_1, *k *_denotes all the different positions of the atoms of *a*_*k*_. Then, we connected all pairs with *d*(*a*_1_, *a*_2_) < 5^*o*^*A *by an edge.

### Calculating global centrality measurements

The *closeness centrality*, the *betweenness *and the *eccentricity *are based on shortest paths. We used the Dijkstra algorithm for tracing the shortest path between two vertices [21]. Hence, every vertex *p *is assigned a *betweenness *value obtained by counting the number of times each node is traversed in this process. The *closeness centrality cc*_*p *_and the *eccentricity e*_*p *_are obtained by calculating:



where *n *is the number of vertices in the network and *l*(*p*, *q*) is the length of a shortest path between *p *and *q*.

### The hypergeometric distribution

A random selection of amino acids follows a hypergeometric distribution. That is, let *N *be the total number of amino acids of a given protein and let *K *be the number of amino acids that are essential for the protein. If we select randomly *n *amino acids, then the probability *P*(*X *= *k*) of having *k *amino acids that are essential follows a hypergeometric distribution and satisfies , where  are the combinatorial. Therefore, the probability of predicting more than *k *essential amino acids is . The "Random <c" curves are determined the following way: for every *x *= *K/N*, *y *is the smallest number *k/n*, such that *P*(*X *≥ *k*) ≤ *c*. It is also worth noting that the average value of this distribution is equal to *n K/N*.

### Phylogenetic approaches

Protein sequences from 2LZM, 1BTL, 1HIV, 1GVP, 1A2P and the set of 128 PDB proteins were aligned with their homologues using the *Conseq *server [18] or using the alignments provided for these three proteins in the *Hssp *database [22,23].

The parameters used to run the *Conseq *server for 2LZM, 1BTL and 1HIV were: Maximum likelihood method used to calculate the conservation scores, PSI-BLAST E-value = 0.001, maximum number of homologous sequences = 50 and the number of PSI-BLAST iterations = l (except for 2LZM, for which it was 3). Alignments with 15, 50 and 43 unique sequences were analyzed for 2LZM, 1BTL and 1HIV respectively. For 1GVP and 1A2P, we could not run the *Conseq *server only by using the parameters (there were too few homologues identified). Therefore, we used the homologues determined by the *HSSP *alignments (6 homologues for 1GVP and 20 homologues for 1A2P). The conservation score is a number (called color in this website) between 1 and 9. The score 9 refers to the most conserved residues. The conservation score at a site corresponds to the site's evolutionary rate.

Alternatively, the *HSSP *alignments were used to calculate the percentage of conservation of the amino acids.

### Amino acids on the protein's surface/core

The contribution of the surface area of every amino acid to the protein surface area was calculated using the ASC package [24]. The bigger the surface area of an amino acid is, the more exposed it is. If the surface area of an amino acid is 0, it means that this amino acid is completely buried in the core of the protein.

### Evaluating the reliability of the predictions

Every method depends on a cut-off value which is the proportion of predicted amino acids. In order to make a more precise study, we decided to analyse the evolution of the set (that contains between 0% and 100% of the amino acids).

We use the classical notions of sensitivity and specificity to evaluate the reliability of the predictions. Sensitivity is defined as the proportion of truly predicted residues (TP) divided by the number of residues experimentally determined (T) to be essential (Sensitivity = TP/T). Specificity is defined by the ratio of non-predicted essential residues (residues experimentally determined not to be essential (F) – false predicted essential residues (FP)) divided by the number of residues experimentally determined not to be essential (F) (Specificity = (F-FP)/F). The best values are obtained when specificity and sensitivity are equal to 1. However, if we predict no amino acids, the specificity is always equal to 1; similarly, if we predict all the amino acids, then the sensitivity is equal to 1. This means one of these two criteria alone is not sufficient to analyse the reliability of a method. That is why we decided to calculate the following criterion for every method/cut-off:



Using this combined criterion, we sorted the different centrality measurements (Table 1).

We also compare the sensitivity to the proportion of amino acids that are predicted. This is relevant, because the sensitivity (*i.e. k/K *with the previous notations) is larger than the proportion of amino acids that are predicted (*i.e. n/N*) if and only if the method is better than random (*i.e*. the number *k of *truly predicted amino acids is larger than the average value of the distribution *n K/N*).Therefore, we plot the sensitivity versus the proportion of amino acids that are predicted (see Figs 1, 2 and 3) to analyze/compare all the methods with all the different cut-offs. The higher a curve is, the better its associated method is.

## Authors' contributions

BT participated in the conception of the study, carried out the programming, participated in the design of the study and in the drafting of the manuscript. DB participated in the design and coordination of the study and helped to draft the manuscript. GDR initially conceived the study, participated in its design and computer programming, its coordination, and in the drafting of the manuscript. All authors read and approved the final manuscript.
